# The Kush Drug Epidemic in Sierra Leone: Public Health Implications, Socioeconomic Drivers and Health System Responses

**DOI:** 10.1002/puh2.70388

**Published:** 2026-09-29

**Authors:** Alvin Turay, Ahmed Vandy, Uthman Okikiola Adebayo, Marie Ibranatu Kolipha‐Kamara, Augustus Osborne, Michael Lahai

**Affiliations:** ^1^ Faculty of Medicine European University Tbilisi Georgia; ^2^ Faculty of Pharmaceutical Sciences, College of Medicine and Allied Health Sciences University of Sierra Leone Freetown Sierra Leone; ^3^ Department of Medical Laboratory Services Federal University of Health Sciences Teaching Hospital Ila‐Orangun Nigeria; ^4^ Department of Public Health, Faculty of Medicine and Health Sciences Hormuud University Mogadishu Somalia; ^5^ Department of Digital Health Global Health Focus Africa Kigali Rwanda; ^6^ Department of Public Health Daffodil International University Dhaka Bangladesh; ^7^ Institute for Development of the Western Area Freetown Sierra Leone

**Keywords:** Kush, mental health systems, public health emergency, Sierra Leone, substance use disorders, synthetic cannabinoids, West Africa

## Abstract

**Background:**

Kush, a synthetic drug combining cannabinoids and nitazene opioids, has emerged as a major public health concern in Sierra Leone, with reported prevalence and clinical severity increasing markedly as it was first identified. This review examines the health consequences of Kush use, the socioeconomic and structural drivers underpinning its spread, and the adequacy of health system and policy responses across the Mano River Basin.

**Methods:**

A narrative review was conducted using structured searches of PubMed/MEDLINE, Scopus, Web of Science and Google Scholar (2016–2025). Grey literature, including government policy documents, reports from international organisations, civil society publications and credible media sources, was systematically reviewed. Eligible sources addressed Kush or related synthetic drug use in Sierra Leone and the wider West African subregion, with emphasis on health outcomes, socioeconomic determinants, health system capacity and policy responses.

**Results:**

Kush exhibits unpredictable toxicity and high dependence potential, with documented associations including acute psychosis, cognitive impairment, severe morbidity and rising mortality. Weak surveillance systems likely underestimate its burden. Structural drivers include entrenched youth unemployment, poverty, rapid urbanisation, post‐conflict trauma and limited access to education and mental health services. Regional dynamics across Sierra Leone, Liberia, Guinea and The Gambia reinforce its transnational spread. Responses since 2024 have intensified but remain disproportionately focused on law enforcement, whereas prevention, treatment and workforce development remain critically under‐resourced.

**Conclusion:**

The Kush crisis exemplifies a complex, cross‐border public health emergency. Addressing it requires integrated, evidence‐informed strategies that strengthen health systems, expand community‐based interventions, improve toxicological surveillance and enhance regional coordination under ECOWAS frameworks.

## 1 Introduction

Synthetic drug markets in sub‐Saharan Africa are evolving at a pace that outstrips the capacity of health systems and regulatory infrastructure [1]. Nowhere is this more acutely illustrated than in Sierra Leone, where a substance known locally as Kush has, within the past decade, transformed from a marginal urban drug into a nationwide and increasingly regional public health emergency [1, 2]. In April 2024, the Government of Sierra Leone formally declared Kush a public health emergency, a designation reflecting both the drug's severe clinical profile and the structural deficiencies that have contributed to its spread [3, 4].

Unlike classical drugs of abuse with well‐characterised pharmacology, Kush is a heterogeneous and dynamically formulated substance whose composition varies by source, time and preparation method [1, 5]. Toxicological analyses and law‐enforcement seizure data indicate that Kush is typically produced by saturating a plant‐based carrier, most often cannabis or other dried herbal material, with synthetic cannabinoids and, increasingly, nitazene‐class opioids, the latter being among the most potent and dangerous compounds in the illicit drug landscape [5]. There are also reports suggesting the incorporation of pulverised human bones into Kush. This chemical heterogeneity complicates surveillance, clinical management and regulatory enforcement simultaneously.

The health sequelae are severe and multidimensional. Clinically documented outcomes include acute psychosis, profound cognitive impairment, wasting, cutaneous ulceration, limb oedema and immunosuppression [1, 6, 7]. Psychiatric services in Sierra Leone have recorded thousands of Kush‐related admissions, with a retrospective study finding that 59% of substance‐use‐disorder admissions over 16 months are related to Kush [8]. Mortality is reported but likely undercounted, given the absence of systematic toxicological death surveillance [7]. Beyond clinical endpoints, the crisis has disrupted families, accelerated school dropout, deepened youth unemployment and heightened community insecurity [9, 10, 11].

Despite this growing evidence base, critical knowledge gaps persist. First, there is limited integrated synthesis of the public health burden of Kush that situates it within the regional dynamics of the Mano River Basin. Second, the socioeconomic and structural drivers that sustain its spread, including unemployment, post‐conflict trauma, porous cross‐border trade networks and fragile health systems, have not been examined within a unified analytical framework. Third, although national and regional policy responses have intensified, their effectiveness, operational constraints and implementation fidelity remain poorly documented [12, 13]. These gaps limit the capacity of policymakers, health planners and researchers to design targeted, scalable interventions.

In line with the gaps stated, this narrative review aims to consolidate current evidence on the health consequences of Kush, examine socioeconomic and structural drivers at both national and regional levels, evaluate existing health system responses and their limitations and identify policy and intervention opportunities. Its findings are intended to support health professionals in strengthening clinical preparedness, guide policymakers in designing equity‐sensitive prevention and treatment strategies and orient researchers toward high‐priority evidence gaps in an area where the cost of inaction continues to mount.

## 2 Methods

We employed a narrative review design to examine the public health impact of the Kush drug crisis in Sierra Leone and the wider Mano River Basin, focusing on health outcomes, socioeconomic and structural drivers, and health system and policy responses. A narrative approach was selected over a systematic review given the nascent and heterogeneous nature of the evidence base, which spans qualitative studies, retrospective clinical audits, surveillance reports, policy documents and grey literature. The study was not registered prospectively, consistent with narrative review methodology.

A structured literature search was conducted from January to April 2025 across PubMed/MEDLINE, Scopus, Web of Science and Google Scholar. Search terms were applied in combination: ‘Kush’, ‘synthetic cannabinoids’, ‘nitazenes’, ‘substance use disorders’, ‘mental health’, ‘public health’, ‘health system’, ‘policy response’, ‘Sierra Leone’, ‘West Africa’, ‘Mano River Basin’, ‘Liberia’ and ‘Guinea’. Reference lists of all relevant publications were hand‐searched for additional sources.

Given the limited volume of peer‐reviewed primary literature, grey literature was systematically reviewed, and credibility was assessed on the basis of institutional source, methodological transparency and relevance to the review objectives. The review included 91 literature sources, consisting of 48 (52.7%) peer‐reviewed publications, 30 (33.0%) grey literature sources and 13 (14.3%) media sources, including documents from the Government of Sierra Leone, ECOWAS, the United Nations Office on Drugs and Crime (UNODC), the Global Initiative Against Transnational Organized Crime, Partners in Health and verifiable media outlets. Sources published between 2016 and 2025 were eligible if they addressed Kush or related synthetic drug use in Sierra Leone or neighbouring West African countries and reported on health outcomes, social determinants, health system capacity or policy responses. Only English‐language literature was included. Studies published outside the specified period, unrelated to the review objectives or not addressing the predefined domains were excluded.

Data were extracted using a structured framework and synthesised thematically across four domains: public health impacts and epidemiology, socioeconomic and structural drivers, health system responses and challenges, and policy and intervention opportunities. Formal risk‐of‐bias assessment was not undertaken, given the heterogeneity of source types; however, the quality and provenance of individual sources were considered during synthesis. As only publicly available secondary data were used, ethical approval was not required.

### 2.1 Public Health Implications of the Kush Crisis

#### 2.1.1 Epidemiological Spread and Burden

Kush use, initially concentrated among urban youth in Freetown, has over the past decade spread across Sierra Leone and into neighbouring Guinea, Liberia and The Gambia, representing a transition from a national substance‑use concern to a cross‑border public health crisis [1, 7]. The epidemic is unfolding within health systems that were already strained prior to Kush's emergence, marked by limited mental health and addiction treatment services, weak surveillance capacity and critical workforce shortages [14].

Mortality attributable to Kush is documented across urban and peri‐urban communities, though the true scale of fatalities likely exceeds official reports. Underreporting is structural: Sierra Leone lacks the forensic toxicology infrastructure necessary to confirm drug‐related deaths systematically, and stigma discourages families from disclosing circumstances of death [7]. This surveillance gap means that policy responses are being calibrated against an incomplete picture of the epidemic's lethality.

Hospital admission data offer the most reliable indicator of clinical burden. At the Sierra Leone Psychiatric Teaching Hospital, Kush‐related admissions increased by approximately 4000% between 2020 and 2023, reaching close to 1865 cases over that period [1]. A retrospective study of 719 substance‐use‐disorder admissions over 16 months found that 59% involved Kush [8], suggesting that Kush now accounts for the majority of the clinical workload at the country's principal psychiatric facility. These figures likely still underrepresent demand, given substantial barriers to formal health‐seeking, including cost, geographic distance, stigma and limited awareness of available services.

#### 2.1.2 Clinical Profile: Physical and Neuropsychiatric Morbidity

The clinical presentation of Kush use is severe and multifaceted. Limited understanding of its clinical course and prognosis, combined with inadequate treatment infrastructure, resource constraints and poor continuity of care, poses substantial challenges to effective management.

The neuropsychiatric sequelae are prominent and include acute psychotic episodes, mood dysregulation, significant cognitive impairment and a high‐dependence syndrome that is difficult to manage in settings with limited pharmacological and psychosocial resources [3, 15]. Although current evidence does not provide prevalence estimates for anxiety or depression among Kush users, existing studies have predominantly described acute neuropsychiatric manifestations, including agitation and delirium, whereas the burden of common mental disorders such as anxiety and depression remains insufficiently characterised [15]. During acute intoxication, some individuals may exhibit pronounced drowsiness, reduced responsiveness or episodes of falling asleep while standing [1, 16]. These observable manifestations have contributed to the colloquial characterisation of Kush as a ‘zombie drug’ in some affected communities, although this term is not a clinical descriptor.

Physical morbidity is equally striking. Chronic users develop visible and debilitating somatic complications, including persistent cutaneous ulcers, open lesions and pronounced oedema of the lower extremities [7]. Progressive wasting, severe nutritional deficiency and impaired immune function create conditions in which wounds become infected readily, heal poorly and in advanced cases may significantly restrict mobility and functional independence [1, 6, 7]. These physical manifestations are clinically distinctive and have contributed to widespread public and media attention to the health impact of Kush.

High relapse rates following treatment reflect the combined effects of physical dependence, inadequate rehabilitation infrastructure, the absence of structured aftercare and the persistence of the underlying social conditions that drove initial use [7, 17]. Without continuity of care and reintegration support, individuals are frequently readmitted, adding to the burden on an already strained system.

### 2.2 Societal and Economic Impacts

Reports have suggested that pulverised human bone material may have been incorporated into certain Kush preparations. Although these claims remain unsubstantiated, they heightened public concern in Sierra Leone and contributed to increased national attention preceding the government's declaration of a public health emergency [1].

The harms generated by the Kush crisis extend well beyond direct users. At the household level, addiction is associated with income loss, family fragmentation, domestic conflict and the displacement of young people into homelessness [18, 19]. Children in affected families face elevated risks of educational disruption, neglect and emotional harm. At the community level, rising rates of theft and drug‑related gang activities pose risks to public safety and contribute to social fragmentation [20, 21].

Adolescents and young adults are disproportionately affected, with Kush use linked to school disengagement, dropout, unemployment and progressive social marginalisation. These outcomes create self‑reinforcing cycles of vulnerability and exclusion [9, 10, 11]. Gender disparities compound these effects: Women who use Kush face layered stigma at household and community levels, encounter reduced access to treatment and social support and remain underserved by a response architecture that lacks gender‑responsive care [22, 23].

The macroeconomic burden is also substantial, though poorly quantified. It encompasses lost labour productivity among working‑age youth, increased healthcare expenditure, intensified law‑enforcement costs and broader fiscal consequences of social instability [9]. Rigorous economic assessments of these costs remain absent, representing an important evidence gap that would strengthen the case for sustained investment in prevention and treatment.

#### 2.2.1 Regional Dimensions

Framing the Kush crisis as a national problem risks obscuring the cross‐border dynamics that fundamentally shape its trajectory. The drug's spread into Liberia, Guinea and The Gambia indicates that supply chains, consumption patterns and social norms surrounding Kush are diffusing along existing migration, trade and social networks across the Mano River Basin [7, 24]. Porous land borders, informal cross‐border commerce and established trafficking routes create a regional socioeconomic space in which substances and their associated cultures of use circulate relatively freely [1, 24, 25].

Law enforcement operations at major border crossings, such as Jendema and Bo Waterside between Sierra Leone and Liberia, have intercepted Kush shipments concealed in passenger vehicles and commercial transport, confirming that cross‐border trafficking is systematic rather than incidental [26, 27]. Regional health system responses have not kept pace with these transnational dynamics, and the absence of harmonised surveillance, data‐sharing and treatment protocols across affected countries significantly limits the effectiveness of national‐level interventions [24, 28].

### 2.3 Socioeconomic and Structural Drivers of Kush Use

#### 2.3.1 Youth Unemployment and Economic Exclusion

Structural economic conditions are among the most potent and proximate drivers of Kush use across the Mano River Basin. Sierra Leone, Liberia and Guinea all face high rates of youth unemployment compounded by the concentration of economic activity in capital cities, an expanding population of young urban migrants and few formal employment pathways [29, 30]. In this context, substance use can emerge both as a coping mechanism for psychological distress and as a social phenomenon embedded in peer networks defined by economic frustration and limited prospects for upward mobility [6, 29].

Informal settlements in Freetown, where young men in particular face acute economic exclusion, have been identified as epicentres of Kush use and distribution [8, 31, 32]. Analogous dynamics have been documented in post‐conflict Liberia, where informal labour markets, fragile peri‐urban economies and limited vocational alternatives have created comparable risk environments [33, 34]. In these settings, the low cost of Kush appears to make it particularly accessible to economically vulnerable youth populations, although the extent to which pricing reflects deliberate market strategies by suppliers has not been systematically documented.

#### 2.3.2 Cross‐Border Mobility and Trafficking Networks

Population mobility substantially reinforces the regional character of the Kush crisis. Long‐standing patterns of cross‐border movement for mining, agricultural labour, petty trade and family ties have created a shared socioeconomic space in which new drug forms and consumption practices diffuse rapidly [28, 35]. Border communities function less as discrete national units than as interconnected hubs through which substances, cultural practices and economic transactions circulate continuously [24, 25].

Kush production and distribution are embedded within these cross‐border informal economies. Supply networks span multiple countries, exploiting established trade routes and migration corridors [7, 24]. The interconnected trade and social ties linking Sierra Leone with both Liberia and Guinea, ties that have historical roots in shared pre‐colonial identities, wartime displacement and post‐conflict reconstruction labour markets, facilitate the physical movement of substances and the social diffusion of urban drug cultures that normalise their use [24, 36, 37].

#### 2.3.3 Post‐Conflict Trauma and Social Disruption

Sierra Leone, Liberia and Guinea each carry the enduring social and psychological legacy of civil conflict. Research on post‐conflict populations demonstrates that exposure to violence, displacement and loss substantially increases the risk of substance use, particularly among young people who lack access to psychosocial support, mental health services and stable social networks [38, 39]. For many users, Kush may function as self‐medication for untreated trauma, anxiety and depression, conditions that are prevalent but largely unaddressed in contexts with minimal mental health infrastructure.

The erosion of family and community structures in the aftermath of conflict further reduces the protective factors that buffer against substance use initiation and escalation [40, 41]. When parental supervision, community monitoring and traditional mentorship systems are weakened, young people already navigating economic insecurity and social marginalisation become substantially more vulnerable to drug use and to the peer and environmental pressures that sustain it [42, 43].

#### 2.3.4 Health System Structural Weaknesses

Critical structural deficiencies in West African health systems create permissive environments in which substance use disorders develop and persist without adequate response. Mental health and addiction services across the region remain chronically underfunded, geographically centralised and insufficiently staffed [44, 45]. Referral pathways are poorly coordinated, resulting in fragmented care and weak continuity for individuals requiring long‐term management [46, 47]. Limited toxicological laboratory capacity and inadequate pharmacovigilance infrastructure further impede detection and monitoring of emerging psychoactive substances, hampering both clinical response and policy development [46, 48, 49].

These structural limitations are mutually reinforcing and produce a system that is reactive rather than preventive, capable of responding inadequately to acute presentations but unable to systematically identify, engage or retain individuals with substance use disorders across the full care continuum. The result is a substantial and growing unmet treatment need that, without structural reform, will continue to expand in proportion to the crisis.

### 2.4 Health System Responses and Challenges

#### 2.4.1 Regional Mental Health System Gaps

The Kush epidemic has laid bare persistent structural deficiencies in mental health systems across West Africa. Services remain underfunded, centralised in capital cities, and reliant on a small number of specialist facilities that are poorly equipped to meet demand for substance use treatment [50, 51]. Across Sierra Leone, Liberia and Guinea, mental health investment as a proportion of overall health expenditure falls well below internationally recommended thresholds, creating systems that are unable to respond effectively to either routine demand or emergent crises [44, 45, 52].

Sierra Leone, widely regarded as the epicentre of the Kush crisis, exemplifies these regional weaknesses in sharp relief. The national mental health workforce comprises only two specialist psychiatrists, two clinical psychologists, 19 mental health nurses, and a single pharmacist for a population of approximately eight million people [14]. Psychiatric services are delivered through two under‐resourced hospitals concentrated in the capital, leaving rural and peri‐urban districts with no meaningful access to formal mental health care [53, 54]. This geographic concentration of limited expertise creates profound inequities that disproportionately affect the populations most vulnerable to Kush.

The surge in Kush‐related presentations has placed acute pressure on these already fragile services. Patients frequently arrive with complex comorbidities such as concurrent psychosis, anxiety, depression and severe physical complications that exceed the management capacity of facilities lacking adequate staffing, medications, inpatient beds and diagnostic infrastructure [15, 38]. Emergency units and psychiatric wards frequently exceed capacity, diverting resources from other essential health services and creating bottlenecks that delay treatment for non‐Kush presentations [1, 6].

#### 2.4.2 Stigma and Inequitable Access to Care

In many West African cultural contexts, substance use disorders are interpreted not as medical conditions requiring treatment but as moral failures, manifestations of spiritual affliction or evidence of personal weakness. These normative frameworks discourage open disclosure, delay help‐seeking and promote punitive and exclusionary responses rather than therapeutic engagement [55]. The resulting stigma is a significant and independent barrier to treatment access, operating at household, community and institutional levels simultaneously.

Women and young people affected by substance use disorders face compounded stigma that further constrains their engagement with formal services [56, 57]. Existing treatment and rehabilitation programmes in Sierra Leone were designed without explicit attention to gender‐responsive care, leaving women without the specialised support they require. Evidence indicates that women are less likely to present for treatment, less likely to be referred by community health workers and less likely to access the social and economic reintegration support needed for sustained recovery [22, 23]. These gender disparities represent both a moral failure and a public health inefficiency that weakens the overall impact of the response.

#### 2.4.3 Government and Civil Society Responses in Sierra Leone

Following the declaration of a national public health emergency in April 2024, the Government of Sierra Leone established a National Task Force with five strategic priorities: awareness and prevention, care and treatment, social support, law enforcement, and community partnership [58]. Concrete investments followed: the Kissy Rehabilitation Centre was expanded, and a new National Rehabilitation Centre was opened in Hastings to provide detoxification, counselling and structured rehabilitation [59, 60]. The Ministry of Health launched a Mental Health Champions Network aimed at strengthening community‐based mental health awareness and advocacy, including on substance use [61].

These developments represent meaningful progress, particularly given the resource‐constrained context. However, uptake of government‐run rehabilitation programmes has remained modest relative to need; a few hundred individuals accessed services following launch, a fraction of those requiring care [62]. Reports of some government‐funded rehabilitation centres closing temporarily due to funding shortfalls highlight the fragility of these gains and the risk that political commitments will not translate into sustained operational capacity [63].

Civil society and faith‐based organisations have emerged as essential actors in the response, often filling gaps that government services cannot reach. Youth‐led and community‐based organisations run awareness campaigns, provide peer counselling and offer vocational skills training to at‐risk young people. Caritas Freetown has established a dedicated Kush Rehabilitation and Empowerment Centre offering integrated medical care, psychological support and livelihood training [64]. Non‐governmental organisations, including Partners in Health, have contributed counselling capacity, peer‐support networks and stigma‐reduction initiatives targeting youth populations [65, 66]. These civil society contributions are valuable but cannot compensate for systemic underinvestment in public mental health infrastructure.

#### 2.4.4 Governance, Coordination and Surveillance Deficits

Fragmented governance and weak intersectoral coordination among agencies responsible for health, social welfare, law enforcement and education undermine the coherence and impact of the national response [67, 68]. Vertical, siloed institutional structures limit the ability to implement integrated strategies that address the full spectrum of Kush‐related harms, from primary prevention to acute clinical management to long‐term social reintegration.

The absence of robust, standardised surveillance systems for monitoring Kush‐related health outcomes is a particularly acute operational gap [6, 12]. Without reliable epidemiological data on incidence, prevalence, morbidity and mortality, response planning is reactive rather than strategic. Limited toxicological capacity means that the composition of street Kush is poorly characterised in real time, hampering clinical management protocols and early warning systems for particularly toxic drug batches [48, 49]. These surveillance deficits are not unique to Sierra Leone but are shared across the Mano River Basin, making regional coordination difficult in the absence of comparable baseline data.

### 2.5 Policy and Public Health Opportunities

#### 2.5.1 A Regional Framework for Coordinated Action

The transnational character of the Kush crisis demands that policy responses extend beyond national borders to encompass a coordinated regional framework. The rapid spread of Kush across the Mano River Basin, facilitated by porous frontiers, informal commerce and established trafficking networks, means that national‐level interventions will remain insufficient in isolation [1, 24]. A unified response by Sierra Leone, Liberia and Guinea, ideally supported by ECOWAS mechanisms, is needed to help prevent the crisis from further straining a region whose governance and security institutions already face significant pressure [5, 24].

ECOWAS has signalled commitment to collective action against synthetic drug trafficking and abuse, and this political momentum represents an important policy window [13]. Leveraging existing regional structures, including the West African Epidemiology Network on Drug Use (WENDU) and the Pan African Epidemiology Network on Drug Use (PAENDU), to harmonise surveillance data, standardise treatment protocols and coordinate cross‐border prevention campaigns would substantially increase the collective effectiveness of individual country responses [69]. Regional intelligence‐sharing platforms and joint border operations, supported by initiatives such as CONNECT‐Africa and AIRCOP, offer proven frameworks for disrupting transnational trafficking networks that can be adapted to address the Kush supply chain [70].

#### 2.5.2 Integrating Substance Use Services Into Primary Health Care (PHC)

A fundamental structural reform with the potential to dramatically expand treatment reach is the integration of substance use prevention and care services into PHC frameworks. In West Africa, PHC facilities are often the first and, in rural and peri‐urban settings, the only point of contact between vulnerable young people and the formal health system [71, 72]. Embedding screening tools, brief interventions, referral pathways and basic psychosocial support within PHC structures would reduce dependence on specialist services, decrease the stigma associated with seeking help at dedicated psychiatric facilities and normalise substance use treatment as a component of routine care.

This integration is particularly urgent given the severity of specialist workforce shortages across the Mano River Basin. Task‐sharing approaches, systematically training non‐specialist community health workers to deliver evidence‐based mental health interventions under appropriate supervision, offer a pragmatic pathway to expanding service access without waiting for the long‐term pipeline of specialist training to yield results [73, 74, 75]. Ethiopia's experience integrating mental health and substance use services into PHC provides a relevant model from a comparable low‐resource context [72].

#### 2.5.3 Strengthening Surveillance and Toxicological Capacity

Effective public health responses require reliable data. Strengthening regional surveillance and data‐sharing systems is therefore a foundational investment rather than an optional enhancement. Achieving this requires dedicated infrastructure for standardised data collection and secure storage, analytical capacity to interpret trends in real time and toxicological laboratory systems capable of rapidly characterising the evolving composition of street drugs [76, 77]. Expanding network platforms such as WENDU, improving forensic toxicology capacity at national reference laboratories and adopting harmonised reporting systems across health ministries would enable earlier detection of dangerous drug batches, better characterisation of mortality and more strategic allocation of prevention and treatment resources [69, 78, 79].

The African Union's ongoing initiatives to strengthen research and data collection capacity across member states provide a political and institutional foundation for these investments [69, 77]. Linking national drug surveillance systems to regional and global platforms would further enhance the quality and comparability of West African drug epidemiology data, supporting more informed advocacy for sustained resource allocation.

#### 2.5.4 Prevention Through Upstream Social Investment

Addressing the structural conditions that drive Kush use is indispensable to achieving sustainable population‐level impact. Prevention programmes that reduce exposure to drug use without simultaneously addressing youth unemployment, educational exclusion and post‐conflict trauma will, at best, achieve modest and short‐lived effects. Governments across the Mano River Basin should invest in vocational training, youth entrepreneurship programmes and formal employment initiatives targeted at the communities and age groups most vulnerable to Kush initiation [80, 81].

Reintegration support for individuals in recovery is equally critical. Without structured pathways back into education, employment and stable housing, supported by community‐led support groups and sustained psychosocial care, relapse rates will remain high and the investment in treatment will yield diminishing returns [6, 82, 83]. Cross‐border public awareness campaigns, designed to reduce stigma and promote help‐seeking, represent a relatively low‐cost intervention with potential for substantial reach when delivered through community networks, religious institutions and trusted media platforms [84].

#### 2.5.5 Legislative Reform and Public Health Reorientation

Recent legislative and institutional measures in Sierra Leone, including the establishment of a special drug court, a comprehensive review of drug laws, the creation of a dedicated enforcement unit and the proposal of a special community intervention fund, signal genuine political commitment to a sustained response [85, 86, 87]. These are the important steps. However, effective responses ultimately require a fundamental reorientation of the policy framework from one that privileges criminal enforcement toward one that centres public health, harm reduction and the protection of human dignity.

The reform of drug laws to reduce the criminalisation of people who use drugs, diverting individuals toward treatment rather than incarceration, would improve health outcomes, reduce the social harms of imprisonment among an already marginalised population and free enforcement resources to focus on organised trafficking networks and high‐level supply chain actors [88]. Ghana's recent drug policy reforms offer a relevant precedent within the West African context and merit consideration as a regional model [88]. Governments across the Mano River Basin should integrate substance use treatment into national health financing mechanisms, ensuring that treatment and rehabilitation programmes are adequately and sustainably funded irrespective of the political cycle [71, 72, 89].

### 2.6 Limitations

This review has several limitations that should be considered when interpreting its findings. First, the evidence base on Kush remains limited and heterogeneous, drawing heavily on grey literature, media reporting and small or single‐site clinical studies rather than population‐representative epidemiological data; prevalence and mortality figures cited throughout should therefore be regarded as indicative rather than precise estimates of national or regional burden. Second, the chemical composition of Kush is not systematically characterised: toxicological data derive from a small number of forensic analyses of seized samples, and the extent to which these findings generalise across time, location and supply source is uncertain. Third, the narrative review design, chosen given the heterogeneity of the evidence base, does not include formal risk‐of‐bias assessment or systematic quality appraisal of included sources, which may introduce selection bias despite efforts to draw on the most credible and recent material available. Fourth, mortality and hospital admission data likely reflect only a fraction of the true burden, given weak surveillance infrastructure, low rates of formal health‐seeking and stigma‐related underreporting; the direction and magnitude of this bias cannot be quantified with available data. Finally, most cited sources originate from Sierra Leone, and comparative data from Liberia, Guinea and The Gambia remain sparse, which limits the strength of conclusions drawn about the regional dynamics of the crisis. These limitations underscore the need for the standardised surveillance and toxicological monitoring systems discussed above.

## 3 Conclusion

The Kush crisis in Sierra Leone and the wider Mano River Basin is not an aberrant or isolated phenomenon but the symptomatic expression of deep structural vulnerabilities: fragile health systems, entrenched youth unemployment, post‐conflict trauma, unaddressed mental health needs and the exploitation of regional governance gaps by organised criminal networks. Kush, a heterogeneous synthetic drug whose unpredictable toxicity and extreme affordability make it uniquely dangerous among marginalised youth populations, has exposed these vulnerabilities with devastating clarity.

The evidence reviewed here makes clear that effective responses must operate simultaneously at multiple levels. Clinically, health systems need expanded and better‐equipped mental health and addiction services, with substance use care integrated into PHC and supported by trained, well‐supervised community health workers. Epidemiologically, strengthened toxicological surveillance and standardised data systems are prerequisites for strategic response planning and early warning. Structurally, investments in youth employment, education and social reintegration are not peripheral to the health response but central to it: Without addressing the conditions that make young people vulnerable to Kush, downstream clinical interventions will be perpetually overwhelmed.

The declaration of a national public health emergency in 2024 and recent legislative reforms in Sierra Leone represent genuine progress. However, political commitments must translate into sustained, adequately funded operational programmes, not one‐off investments vulnerable to budgetary contraction or shifting governmental priorities. The pattern of rehabilitation centres closing due to funding shortfalls is a warning that good intentions alone are insufficient. What is required is the institutionalisation of the Kush response within national health financing frameworks and the development of legal and regulatory infrastructure that positions treatment above punishment.

Finally, the transnational character of the Kush crisis suggests that regional coordination is likely to be an important component of an effective response, as no single country is well positioned to address a cross‐border drug epidemic through national action alone. Sierra Leone, Liberia and Guinea share borders, trade routes and broadly similar social and economic conditions that appear to sustain Kush use. A coordinated regional approach, harmonising surveillance, treatment protocols, enforcement intelligence and upstream social policies under a strengthened ECOWAS framework, may offer a response better matched to the scale of the problem.

## Author Contributions

**Alvin Turay** and **Ahmed Vandy** conceptualise the review idea. Alvin Turay, **Uthman Okikiola Adebayo**, **Augustus Osborne** and Ahmed Vandy curated the data. Alvin Turay, Augustus Osborne and Ahmed Vandy provided the original draft of the manuscript. Alvin Turay, **Michael Lahai**, Ahmed Vandy, **Marie Ibranatu Kolipha‐Kamara** and Uthman Okikiola Adebayo reviewed the manuscript. All authors have read the content of this article and approved its submission for publication.

## Funding

The authors have nothing to report.

## Ethics Statement

The authors have nothing to report.

## Conflicts of Interest

The authors declare no conflicts of interest.

## Data Availability

Data sharing is not applicable to this article as no datasets were generated or analysed during the current study.
